# Expression profiling of formalin-fixed paraffin-embedded primary breast tumors using cancer-specific and whole genome gene panels on the DASL^® ^platform

**DOI:** 10.1186/1755-8794-3-60

**Published:** 2010-12-20

**Authors:** Monica M Reinholz, Jeanette E Eckel-Passow, S Keith Anderson, Yan W Asmann, Michael A Zschunke, Ann L Oberg, Ann E McCullough, Amylou C Dueck, Beiyun Chen, Craig S April, Eliza Wickham-Garcia, Robert B Jenkins, Julie M Cunningham, Jin Jen, Edith A Perez, Jian-Bing Fan, Wilma L Lingle

**Affiliations:** 1 Department of Laboratory Medicine and Pathology, Mayo Clinic, 200 First St SW, Rochester, Minnesota, 55905, USA; 2 Division of Biomedical Statistics and Informatics Mayo Clinic, 200 First St SW, Rochester, Minnesota, 55905, USA; 3 Department of Pathology, Mayo Clinic, 13400 E. Shea Blvd, Scottsdale, Arizona, 85259, USA; 4 Section of Biostatistics, 13400 E. Shea Blvd, Scottsdale, Arizona, 85259, USA; 5 Department of Scientific Research, Illumina Inc., 9885 Towne Centre Drive, San Diego, California, 92121, USA; 6 Division of Pulmonary and Critical Care Medicine, Mayo Clinic, 200 First St SW, Rochester, Minnesota, 55905, USA; 7 Division of Hematology and Oncology, Mayo Clinic, 4500 San Pablo Road, Jacksonville, Florida, 32224, USA

## Abstract

**Background:**

The cDNA-mediated Annealing, extension, Selection and Ligation (DASL) assay has become a suitable gene expression profiling system for degraded RNA from paraffin-embedded tissue. We examined assay characteristics and the performance of the DASL 502-gene Cancer Panel^v1 ^(1.5K) and 24,526-gene panel (24K) platforms at differentiating nine human epidermal growth factor receptor 2- positive (HER2+) and 11 HER2-negative (HER2-) paraffin-embedded breast tumors.

**Methods:**

Bland-Altman plots and Spearman correlations evaluated intra/inter-panel agreement of normalized expression values. Unequal-variance *t*-statistics tested for differences in expression levels between HER2 + and HER2 - tumors. Regulatory network analysis was performed using Metacore (GeneGo Inc., St. Joseph, MI).

**Results:**

Technical replicate correlations ranged between 0.815-0.956 and 0.986-0.997 for the 1.5K and 24K panels, respectively. Inter-panel correlations of expression values for the common 498 genes across the two panels ranged between 0.485-0.573. Inter-panel correlations of expression values of 17 probes with base-pair sequence matches between the 1.5K and 24K panels ranged between 0.652-0.899. In both panels, *erythroblastic leukemia viral oncogene homolog 2 *(*ERBB2*) was the most differentially expressed gene between the HER2 + and HER2 - tumors and seven additional genes had p-values < 0.05 and log2 -fold changes > |0.5| in expression between HER2 + and HER2 - tumors: *topoisomerase II alpha *(*TOP2A*), *cyclin a2 *(*CCNA2*), *v-fos fbj murine osteosarcoma viral oncogene homolog *(*FOS*), *wingless-type mmtv integration site family, member 5a *(*WNT5A*), *growth factor receptor-bound protein 7 *(*GRB7*), *cell division cycle 2 *(*CDC2*), *and baculoviral iap repeat-containing protein 5 *(*BIRC5*). The top 52 discriminating probes from the 24K panel are enriched with genes belonging to the regulatory networks centered around *v-myc avian myelocytomatosis viral oncogene homolog *(*MYC*), *tumor protein p53 *(*TP53*), and *estrogen receptor α *(*ESR1*). Network analysis with a two-step extension also showed that the eight discriminating genes common to the 1.5K and 24K panels are functionally linked together through *MYC*, *TP53*, and *ESR1*.

**Conclusions:**

The relative RNA abundance obtained from two highly differing density gene panels are correlated with eight common genes differentiating HER2 + and HER2 - breast tumors. Network analyses demonstrated biological consistency between the 1.5K and 24K gene panels.

## Background

Gene expression profiling is a rapidly advancing field and has become a useful tool in clinical oncology to identify molecular differences and similarities that can be correlated with clinical behavior and drug responsiveness. Numerous genes are controlled by complex regulatory networks and are involved in the development and progression of breast cancer, and these genes are the key factors in determining each characteristic of the tumor [1,2]. The resulting gene signatures may then help define cancer subtypes, predict recurrence of disease and response to specific therapies, and be used to analyze oncogenic pathways [3]. Microarray studies in breast cancer research have demonstrated extensive molecular heterogeneity of breast cancer, identifying distinct tumor classifications not evident based on traditional histopathological methods [4,5]. Molecular phenotyping also has produced gene signatures that may help predict risk of recurrence in early-stage breast cancer patients including several commercially available panels, Mammaprint (Agendia, Amsterdam, Netherlands), OncoType Dx (Genomic Health, Redwood City, CA), and THEROS H/I (HOXB13:IL17BR; bioTheranostics, San Diego, CA) [6-9].

Formalin-fixed, paraffin-embedded (FFPE) tumor samples are routinely used for clinical diagnostic purposes and are the most widely available materials for which patient outcomes are known. However, many microarray-based analyses use intact ribonucleic acid (RNA) from fresh frozen tissue, not a commonly available source of tissue. Thus, FFPE tissue is an invaluable resource for cancer research, particularly for phase III adjuvant clinical trials. These large clinical sample sets are critical for validating molecular profiles of tumor classification, treatment response, and clinical outcome prediction. Although RNA isolated from FFPE is usually highly degraded posing several challenges for microarray based gene-expression profiling, a reverse transcriptase/polymerase chain reaction (RT-PCR)-based microarray technology has been developed to allow high-throughput profiling of paraffin block tissue samples [10-15].

The complementary DNA-mediated Annealing, extension, Selection and Ligation (DASL^®^) assay (Illumina; San Diego, CA) is a gene expression profiling system suitable for use with degraded RNAs such as those derived from FFPE tumor samples [10-18]. The DASL assay resembles RT-PCR and is designed to target small cDNA sequences spanning only 50 bases. This is especially useful for RNA extracted from FFPE tissues as RNA transcripts are typically less than 200 nucleotides in length. The DASL assay monitors gene expression in parallel in archival samples using a minimal amount of total RNA (~200 ng total RNA per assay). Comparable results in sensitivity, reproducibility, and accuracy have been observed between FFPE and snap-frozen tissue of the same tumor when the DASL assay is performed according to the pre-analytic quality control criteria [10-15]. The 502 Cancer Panel^v1 ^(1.5K) and the whole genome panel (WG; 24,526 probes; 24K) are DASL based, commercially available (Illumina, San Diego, CA), and designed specifically for use with FFPE tissue.

The primary objective of this study was to examine the performance of the 1.5K and 24K DASL gene panels to determine whether genes behave similarly between gene panels with differing densities. The primary technical objectives were to evaluate the 1) intra-panel agreement of normalized expression values for technical replicates, 2) intra-panel agreement of normalized expression values for repeated extracts, 3) inter-panel agreement of normalized expression values for the 17 probes from the 1.5K panel that had an exact base-pair sequence match with a sequence internal to a 24K probe and 4) inter-panel agreement of normalized expression values for the 498 genes in common between the two panels. Secondary analyses included biological objectives evaluating the differential gene expression patterns between human epidermal growth factor receptor 2-positive (HER2+) and HER2-negative (HER2-) breast tumors and pathway networks of the highly discriminating genes obtained from the two panels.

## Methods

### Specimens

This study was approved by the Mayo Clinic Institutional Review Board and performed in accordance with institutional and federal guidelines. Informed consent was documented. Twenty archived FFPE breast tumor specimens (procured between 1998 and 2006) were obtained from the Mayo Clinic Tissue Registry, Rochester, MN and were frequently matched on estrogen and progesterone receptor, tumor size, nodal status, and subject age (Table 1). The majority of the tumors were procured before routine and standardized HER2 testing (e.g., HercepTest; Dako, Carpinteria, CA) [19] thus, different immunohistochemical staining techniques were used to determine the HER2 status of the breast tumors in the Tissue Registry, and a HER2 immunohistochemistry (IHC) score of 3+ was defined as > 10% of cells with complete membrane staining according to Food and Drug Administration (FDA)-approved guidelines [20] (Table 1, initial HER2 score). As there have been changes in pathologic interpretation of HER2 expression over time [19], we performed the HercepTest on a fresh cut FFPE tissue section from each specimen according to manufacturer's instructions (Dako). This clinical variable was defined as the HercepTest HER2 score (Table 1), and an IHC score of 3+ was defined as > 30% of cells with complete membrane staining according to the 2007 American Society of Clinical Oncology/College of American Pathology (ASCO/CAP) guidelines [19]. Two pathologists (B.C.; A.E.M.) reviewed the HER2 staining of the tumors on whole sections (B.C.) and tissue microarray sections (A.E.M.) and were 100% concordant, except in the two cases that were re-classified as 2+; the whole section score was 2+ and the average TMA score was 1+. An additional case was classified as 3+ upon initial and central review but the average TMA score was 2+. For HER2 expression level comparisons, tumors with 2+ or 3+ IHC staining patterns in whole sections were considered HER2+ (n = 9) and tumors with 0 or 1+ staining patterns were considered HER2- (n = 11). Of the 20 tumors, 80% (16/20) of the tissues had ≥ 80% invasive tumor component. The clinicopathological characteristics of the 20 tumors are shown in Table 1.

**Table 1:** Clinicopathological characteristics of 20 breast tumors.

Characteristic	Total N = 20	%	HER2+ N = 9 (%)	HER2- N = 11 (%)
Median age (range)	58.5 (35, 76)		57 (35, 73)	59 (38, 76)
Age Group:				
< 40	3	15	2 (22)	1 (9)
40-49	2	10	1 (11)	1 (9)
50-59	6	30	2 (22)	4 (36)
≥ 60	9	45	4 (44)	5 (46)
Initial HER2 Status:				
0	6	30		
1 +	1	5	NA	NA
2 +	0	0		
3 +	13	65		
Hercept Test HER2 Status:				
0	4	20		
1 +	7	35	NA	NA
2 +	2	10		
3 +	7	35		
Estrogen Status:				
Positive	20	100	9 (100)	11 (100)
30% ER+ cells			0 (0)	0 (9)
70% ER+ cells			1 (11)	0 (0)
80% ER+ cells			2 (22)	1 (9)
90% ER+ cells			2 (22)	4 (36)
95% ER+ cells			2 (22)	2 (18)
100% ER+ cells			2 (22)	2 (18)
% cell not recorded			0 (0)	0 (0)
Negative	0	0		
Progesterone Status:				
Positive	19	95	9 (100)	10 (91)
Negative	1	5	0 (0)	1 (9)
Nodes Positive:				
1-3	10	50	5 (56)	5 (46)
4-9	6	30	2 (22)	4 (36)
≥ 10	4	20	2 (22)	2 (18)
Predominant Tumor Histology:				
Ductal	20	100	9 (100)	11 (100)
Lobular	0			
Nottingham Grade:				
2	11	55	4 (44)	8 (73)
3	9	45	5 (56)	3 (27)
Stage:				
2	12	60	5 (56)	7 (64)
3	8	40	4 (44)	4 (36)
Pathologic Tumor Size:				
< 2 cm	5	25	3 (33)	2 (18)
≥ 2 cm	15	75	6 (67)	9 (82)
Year of Block Procurement:				
1998-2000	3	15	22 (22)	1 (9)
2001-2003	11	55	4 (44)	7 (64)
2004-2006	6	30	3 (33)	3 (27)

### RNA Extraction

Total RNA was extracted from six 5 μm thick whole tissue sections from each sample using the Roche Hi-Pure RNA Extraction kit according to manufacturer's instructions (Roche, Indianapolis, IN). The concentration of the purified RNA was determined using a NanoDrop ND-1000 spectrophotometer (NanoDrop Technologies; Wilmington, DE). Purified total RNA samples were stored frozen at -80°C until needed for quality control (QC) analysis and subsequent gene expression profiling. The average RNA yield was 5.4 ± 2.5 μg (range: 2.6-12.6) with an average 260:280 ratio of 2.08 ± 0.05 (range: 1.99-2.22). Representative Agilent tracings demonstrated that the majority of the RNA transcripts were ~ 200 bp in length (data not shown). Replicate extractions were performed for eight of the twenty breast tumors. Additionally, technical replicates were performed on eight tumor specimens including four of the extract replicate samples.

### 1.5K and 24K Gene Panels

Two Illumina human gene panels with partially overlapping probe content were used for the array hybridization experiments. The 502-gene Cancer Panel^v1 ^(1.5K) has 1506 probes associated with 502 unique gene symbols [21]. The whole-genome 24,526 probe (24K) panel [16] was developed based on content derived from the National Center for Biotechnology Information Reference Sequence [NCBI RefSeq Database (Build 36.2, Release 22)]. The 24K has 24,526 probes associated with 18,401 gene symbols. The 1.5K panel has 3 probes representing each gene and the 24K platform has one to eight probes representing a particular gene.

The 498 genes in common between the two panels were determined by matching the gene symbol, gene symbol alias, Reference Sequence (RefSeq) Accession Number, and/or the Entrez Gene ID. Probes from 466 genes were matched by exact gene symbol matches. Probes from an additional 27 genes were matched by their RefSeq Accession Number. Probes from five genes were matched based on gene symbol aliases, Entrez Gene identification numbers, and alternative RefSeq Accession Number. The data discussed in this publication have been deposited in NCBI's Gene Expression Omnibus [22] and are accessible through GEO Series accession number GSE25234 http://www.ncbi.nlm.nih.gov/geo/query/acc.cgi?acc=GSE25234.

### RNA Labeling and Hybridizations

For the 1.5K DASL multiplex experiments, labeling and hybridizations were performed as previously described [11-13] in the Mayo Clinic Genotyping Shared Resource (Rochester, MN). Briefly, 200 ng total RNA was converted to complementary deoxyribonucleic acid (cDNA) using biotinylated oligo-dT_18 _and random nonamer primers, followed by immobilization to a streptavidin-coated solid support. Pre-qualification of cDNA (1 μl) was assessed by quantitative RT-PCR analysis of the housekeeping gene, RPL13a, following the Illumina recommended qPCR. The range of observed Cq values was 19.75-33.34. The biotinylated cDNAs were then annealed to assay-specific oligonucleotides creating PCR templates that were amplified using fluorescently-labeled and biotinylated universal primers. The labeled PCR products were then captured on streptavidin paramagnetic beads, washed and denatured to yield single-stranded fluorescent molecules which were hybridized, via short ~22 nucleotide sequences, to a Sentrix Universal-96 Array Matrix (SAM) for 16 hr using a 60°C to 45°C temperature gradient. For the 24K experiment, the procedure was essentially similar to that described for the 1.5K DASL experiments, the difference being that the assay-specific oligonucleotide designs varied such that the hybridizations were performed via 50 nucleotide sequences to whole-genome gene expression BeadChips (HumanRef-8 v3 Beadchip, Illumina) for 16 hr at 58°C. The 24K experiment was performed at Illumina using aliquots of the same RNA used in the 1.5K experiment. For both the 1.5K and 24K chips the fluorescence intensities were read on BeadArray Readers.

### Analysis of Array Image Data

The probe intensity values were extracted from the images by the GenomeScanner Software within BeadArray Readers. The Gene Expression module from Illumina BeadStudio analysis software was used to process the intensity data and provide a preliminary analysis and measures of quality control. Each oligonucleotide probe is represented, on average, by 30 beads per hybridized sample. BeadStudio summarizes the pixel intensities for each bead and then averages over the redundant beads to associate intensity with each of the unique probes. A number of control oligonucleotide probes are spiked into the hybridization mix to estimate image intensity due to non-specific binding and target binding specificity. Universal Human Reference RNA (UHRR) samples (Agilent Technologies, Santa Clara, CA) comprised from 10 human cell lines were used as control RNA samples to assess the quality of RNA labeling and hybridization in each DASL assay. The UHRR was selected as one of the two standards in the FDA led Microarray Quality Control (MAQC) project [23]. Inter-plate controls were also included to assess reproducibility between plates. For the 24K data, all samples passed array quality control, having robust signal intensities (> 700 counts), good sensitivity (~14000 probes per sample detected (p < 0.01) and having good reproducibility for expression profiles across technical replicates (average r^2^~0.98). For both platforms, the annealing and hybridization controls performed well, indicating that both the DAP annealing (sample-dependent) and array hybridization (sample-independent) components of the assay performed well. In addition, no significant associations (at p < 0.05 level) were observed between the year the block was procured and RPL13a qPCR Cq values or the scanner P95 readings (Additional File 1, Figures S1-S2).

### Statistical Analysis

The non-background corrected expression values were exported from BeadStudio and normalized using fastlo [24], a model-based, intensity-dependent normalization method that produces results essentially the same as those from cyclic loess [25]. Intra- and inter-panel agreement of normalized base-2 logarithm-transformed expression values was evaluated using Bland-Altman plots and Spearman correlations. A Bland-Altman plot is a plot of the difference between two measurements (A - B) against the average of the two measurements (A + B)/2. In comparison to a simple correlation plot of A versus B, a Bland-Altman plot provides a better visualization of the magnitude of disagreement (error and bias) and better highlights outliers and trends in the disagreement. If the differences between two measurements are not related to the magnitude of either measurement, then it is expected that the data will be randomly scattered around the zero horizontal reference line. A local regression line is included on the Bland-Altman plot to visualize trends in the data. Unequal-variance *t*-statistics were utilized from the first extract and first replicate (for patients with technical and/or extract replicates) to test for differences in expression levels between HER2+ and HER2- patients at the probe level. Probes with a p-value < 0.01 and log2-fold change > |1.0| were classified as candidates for being differentially expressed. Next, expression data were summarized for each of the 498 genes in common between the 1.5K and 24K panels by averaging the base-2 logarithm-transformed normalized expression values for all probes that match to a particular gene symbol on the corresponding panel. Unequal-variance *t*-statistics were utilized to test for differences in expression levels between HER2+ and HER2- patients at the gene level; genes with a p-value < 0.05 and log2-fold change > |0.5| were classified as candidates for being differentially expressed. The proportion fold change agreement was analyzed by calculating the proportion of genes that had the same fold change direction; for example, the fold change for a specific gene would be considered in agreement if the fold change values from the 1.5K and 24K were both either positive or negative. All analyses were conducted using the software R [26].

### Network Analysis

Network analyses were performed using MetaCore network building tools (GeneGo Inc., St. Joseph, MI). The Dijkstra's shortest paths algorithm [27]) was used to find the shortest directed paths between the genes allowing two steps in the path. We used the curated interactions only between genes from MetaCore database of interactions. In order to investigate the functional relationships between the top discriminating genes from the 24K panel, probes having p-values < 0.01 and log2-fold change > |1.0| between HER2+ and HER2- tumors were selected for network analysis based on the pair-wise regulatory relationships annotated by MetaCore. Network analysis was also performed on the eight genes that were differentially expressed between HER2+ and HER2- tumors (p-value < 0.05 and log2-fold change > |0.5|) common to both the 1.5K and 24K panels.

## Results

### Inter-panel agreement across the 1.5K and 24K Gene Panels

**Figure 1:**
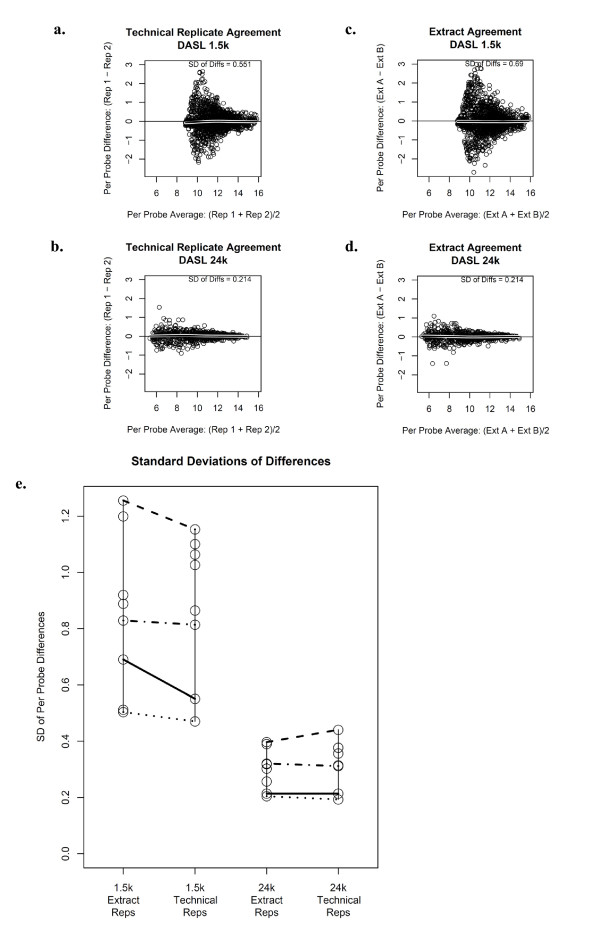
**Bland-Altman plots displaying intra-panel agreement of technical and extract replicates**. **a**. Technical-replicate agreement of normalized expression for the 1.5K panel. **b**. Technical-replicate agreement of normalized expression for the 24K panel. **c**. Extract-replicate agreement of normalized expression for the 1.5K. **d**. Extract-replicate agreement of normalized expression for the 24K panel. The vertical axis denotes the difference in expression values between the technical replicates and the horizontal axis denotes the average expression for each of the 498 genes in common. A local regression line is superimposed on figures a-d. **e**. Summarized standard deviations of the differences for each of the eight samples with technical replicates and eight samples with extract replicates.

The Pearson correlations associated with technical replicates ranged from 0.815 to 0.956 for the 1.5K panel and 0.986 to 0.997 for the 24K panel for the 498 genes in common across the panels. Figures 1a and 1b show Bland-Altman plots comparing the agreement of normalized gene expression intensities across a set of technical replicates associated with a single representative tissue sample for the 1.5 K (**1a**) and 24K (**1b**) panels. For the tissue sample displayed in Figure 1, the variability associated with technical replicates is larger for the 1.5 K panel in comparison to the 24K panel; the standard deviation associated with the difference in expression between the set of technical replicates shown is 0.551 for the 1.5K panel and 0.214 for the 24K panel. The Pearson correlations associated with extract replicates ranged from 0.793 to 0.949 for the 1.5K panel and 0.988 to 0.997 for the 24K panel for the 498 genes in common. Figures 1c and 1d show Bland-Altman plots comparing the agreement of normalized gene expression intensities across extract replicates associated with a single representative subject. For the subject displayed, the variability associated with extract replicates is larger for the 1.5K panel in comparison to the 24K panel; the standard deviation associated with the difference in expression between extract replicates for the same tumor sample is 0.69 for the 1.5K panel and 0.214 for the 24K panel. Figure 1e summarizes the standard deviation associated with the difference in expression across technical replicates for all 8 tissue samples that had technical replicates performed. The standard deviation for technical replicates was notably smaller for the 24K panel indicating that the 24K panel produces more precise expression values. Figure 1e also summarizes the standard deviation associated with the difference in expression across extract replicates. The standard deviation for extract replicates was again notably smaller for the 24K panel indicating that the 24K panel produces more precise expression values. Note that four samples had both technical replicates and replicate extracts and these samples are connected with horizontal lines.

**Figure 2:**
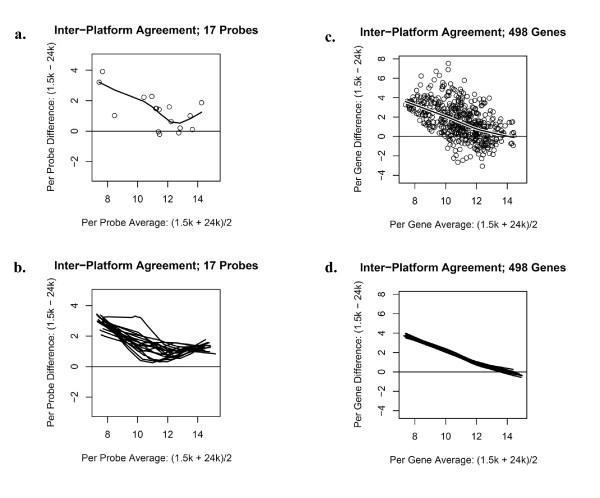
**Bland-Altman plots displaying inter-panel agreement of common probes**. **a**. Inter-panel agreement for the 17 sequence-matched probes for a single representative sample, with a local regression line superimposed. **b**. A local regression line representing each of the 20 samples. **c**. Inter-panel agreement for the 498 gene symbols in common across the 1.5K and 24K panels for a single sample, with a local regression line superimposed. **d**. A local regression line for each of the 20 samples.

Figure 2a is a Bland-Altman plot displaying the inter-panel agreement of the normalized expression values for the 17 sequence-matched probes associated with a single representative tissue sample. Similarly, Figure 2b provides a Bland-Altman plot with a local regression line for each of the 20 samples that were hybridized on both gene panels. In general, the local regression lines are above the zero horizontal reference line indicating that the 1.5K panel produced larger expression values than the 24K panel. More strikingly, the difference in expression was attenuated for probes expressed at a low level. The Pearson correlations associated with the 17 sequenced-matched probes between the 1.5K and 24K panels ranged from 0.652 to 0.899 across the 20 samples. Figures 2c and 2d show Bland-Altman plots displaying the agreement of the normalized expression values for the 498 genes in common across the two platforms. Again, the 1.5K panel generally produced larger expression values than the 24K panel and the difference in expression was attenuated for genes expressed at a low level. The Pearson correlations associated with the 498 common genes between the 1.5K and 24K panels ranged from 0.485 to 0.573 across the 20 samples.

### HER2 expression

**Figure 3:**
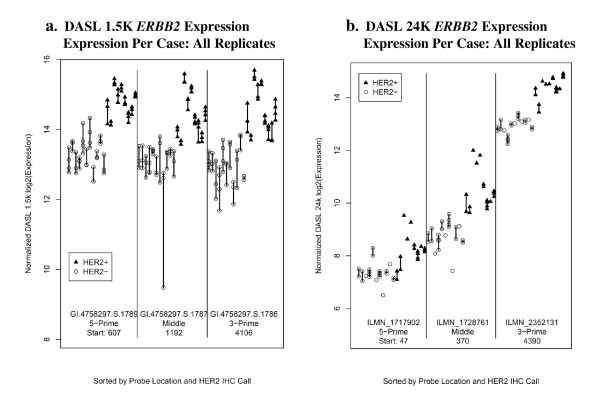
**ERBB2 gene expression.** a. ERBB2 (HER2) expression for the three probes on the 1.5K panel. b. ERBB2 (HER2) expression for the three probes on the 24K panel. From left to right the three probes are ordered from the 5-prime to 3-prime end of ERBB2. The ERBB2 probe positions for the 1.5K panel are: 607 for GI.4758297.S.1789-5prime; 1192 for GI.4758297.S.1787-middle; and 4106 for GI.4758297.S.1786-3prime. The ERBB2 probe positions for the 24K panel are: 47 for ILMN_1717902-5prime; 370 for ILMN1728761-middle; and 4390 for ILMN_2352131-3prime. Internal to each probe the samples are sorted by HER2 IHC 0-3+ values. Open circles represent tumors with HerceptTest IHC scores of 0-1+ and closed triangles represent tumors with HerceptTest IHC scores of 2-3+.

Three probes on the 1.5K panel represent erythroblastic leukemia viral oncogene homolog 2 (*ERBB2*; *HER2*): GI.4758297.S.1789, GI.4758297.S.1787, and GI.4758297.S.1786. Similarly, three probes on the 24K panel represent *ERBB2*: ILMN_1717902, ILMN_1728761, and ILMN_2352131. Figure 3 displays the normalized expression for the *ERBB2 *probes for both panels; probes are plotted in the order of 5-prime, middle, and 3-prime end of the gene. For both panels, *ERBB2 *gene expression was significantly lower in HER2- than in HER2+ samples for all three probes on both panels (p < 0.003 for all probes). The normalized expression across probes was more variable within the 24K panel compared to the 1.5K panel.

At the gene level, larger correlations between the 1.5K and 24K panels were observed for genes that were represented by more probes. For the 498 genes in common across the two platforms, the 1.5K panel had 3 probes representing each gene. However, the 24K panel had 333 genes that were represented by a single probe, 86 that were represented by two probes, 64 that were represented by three probes, and 15 that were represented by four-six probes. The average expression was calculated for each platform for all 498 genes in common. The median correlation of expression levels across the gene panels were 0.36 (1^st^/3^rd ^quartiles: 0.1843/0.5990), 0.46 (1^st^/3^rd ^quartiles: 0.2404/0.6517), 0.49 (1^st^/3^rd ^quartiles: 0.2323/0.6893), and 0.53 (1^st^/3^rd ^quartiles: 0.3915/0.6102) for genes having one, two, three, and four to six probes per gene on the 24K panel, respectively (Additional File 1, Figure S3).

### Fold change agreement of genes differentiating HER2+ vs HER2- tumors

**Figure 4:**
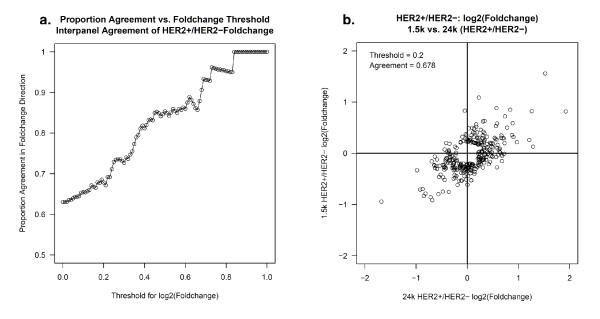
**Fold change agreement of genes differentiating HER2+ versus HER2- tumors**. **a**. Agreement of fold change estimates for the 1.5K and 24K panels for a range of noise thresholds. **b**. Agreement of fold change using a noise threshold of |0.20|, i.e., considering only genes that have a log2-fold change large than |0.20| in at least one of the panels.

In addition to comparing normalized expression values across the two panels, we also evaluated the agreement with respect to fold change estimates of HER2+ versus HER2- expression at the gene level (Figure 4). To determine agreement, the fold change values were dichotomized as follows: a gene was classified as up-regulated if the log2 fold change was larger than zero and down-regulated if the log2 fold change was less than zero. The two panels agreed if both panels called a gene up-regulated or both panels called a gene down regulated. As genes that are expressed at very low levels are usually below the noise threshold and thus, will be randomly classified as up- or down-regulated, it is more appropriate to evaluate agreement among the genes that are expressed above a noise level. To obtain a more accurate estimate of agreement, Figure 4a provides the proportion of concordant calls using a range of noise thresholds. For example, using a noise threshold of zero (i.e., no threshold), the agreement across all 498 common genes was 63% (314/498). The agreement improved to 68% (196/289) and 85% (64/75) when considering only the set of genes that had a log2 fold change (noise threshold) > |0.2| and |0.5| in at least one of the panels, respectively. Figure 4b displays the agreement of expression values using a noise threshold of |0.2|, i.e. considering only genes that produced a log2 fold change larger than |0.2| in at least one of the panels. There were 289 genes where at least one of the panels had a log2 fold change greater than |0.2|; the direction of the fold change was concordant for 97 up-regulated and 99 down-regulated genes and discordant for 93 genes.

Table 2 indicates that the *ERBB2 *gene was the most differentially expressed gene for both panels (p < 0.0001). Seven additional genes had p-values < 0.05 and log2-fold change > |0.5| gene expression change between HER2+ and HER2- tumors in both panels: *topoisomerase II alpha *(*TOP2A*), *cyclin a2 *(*CCNA2*), *v-fos fbj murine osteosarcoma viral oncogene homolog *(*FOS*), *wingless-type mmtv integration site family, member 5a *(*WNT5A*), *growth factor receptor-bound protein 7 *(*GRB7*), *cell division cycle 2 *(*CDC2*), *and baculoviral iap repeat-containing protein 5 *(*BIRC5*). An additional 14 and 17 genes from the 1.5K and 24K panels, respectively, had log2-foldchange > |0.5| and p-values < 0.05; Table 2 provides the log2-fold change and p-values for both panels for these discordant genes.

**Table 2:** Genes that had a log2-fold change > |0.5| and a t-test p-value < 0.05.

	DASL 1.5K		DASL 24K	
Symbol	p-value	log2-fold change	p-value	log2-fold change
*ERBB2*	0.00002	1.560	0.00001	1.522
*TOP2A*	0.00238	0.820	0.02047	1.260
*CCNA2*	0.00275	0.880	0.03336	0.572
*FOS*	0.01719	-0.834	0.01814	-0.848
*WNT5A*	0.01779	-0.697	0.01086	-0.873
*GRB7*	0.01802	0.816	0.00027	1.924
*CDC2*	0.02282	0.588	0.00946	0.886
*BIRC5*	0.03098	0.721	0.04624	0.738
*MST1R*	0.00142	-0.855	*0.18625*	*-0.715*
*CTSL1*	0.00504	0.565	*0.46187*	*0.173*
*KLF6*	0.00629	-0.530	*0.62433*	*0.078*
*VAV2*	0.00873	-0.612	*0.98142*	*0.005*
*ELK3*	0.00877	-0.541	*0.31326*	*-0.161*
*AREG*	0.01172	1.089	*0.04873*	*0.227*
*RASA1*	0.01268	0.831	*0.70107*	*-0.048*
*LIG4*	0.01291	-0.526	*0.66921*	*0.078*
*XRCC2*	0.01457	0.622	*0.02547*	*0.307*
*RAD52*	0.01908	-0.530	*0.60233*	*-0.117*
*IGF2*	0.02324	-0.748	*0.02119*	*-0.477*
*TFAP2C*	0.03411	0.633	*0.01484*	*0.339*
*TYMS*	0.04853	0.582	*0.06550*	*0.582*
*TNFSF10*	0.04884	0.533	*0.06095*	*0.562*
*CDKN2D*	*0.33534*	*0.190*	0.00431	0.501
*IGFBP6*	*0.05152*	*-0.289*	0.00447	-0.627
*BCL6*	*0.00786*	*-0.417*	0.00866	-0.520
*CCNE1*	*0.86111*	*0.036*	0.01106	0.629
*BMP4*	*0.06932*	*-0.786*	0.01395	-0.815
*PTCH1*	*0.64341*	*-0.122*	0.01807	-0.589
*PBX1*	*0.13206*	*0.276*	0.01853	0.550
*EGR1*	*0.09200*	*-0.232*	0.01887	-0.646
*RAB8A*	*0.88338*	*-0.027*	0.02049	0.603
*BLM*	*0.24745*	*0.355*	0.02160	0.875
*MYCN*	*0.45713*	*0.262*	0.02168	1.213
*MPL*	*0.00606*	*-0.460*	0.02810	-0.551
*TGFB2*	*0.29992*	*-0.332*	0.03025	-0.979
*RAD54L*	*0.94663*	*0.013*	0.03996	0.642
*MAP3K8*	*0.73556*	*-0.050*	0.04151	0.683
*CDC25C*	*0.47633*	*0.197*	0.04316	0.728
*HMMR*	*0.12330*	*0.502*	0.04816	0.779

**Note**: Italicized values do not pass the p-value or fold change threshold.

### Network Analyses

**Figure 5:**
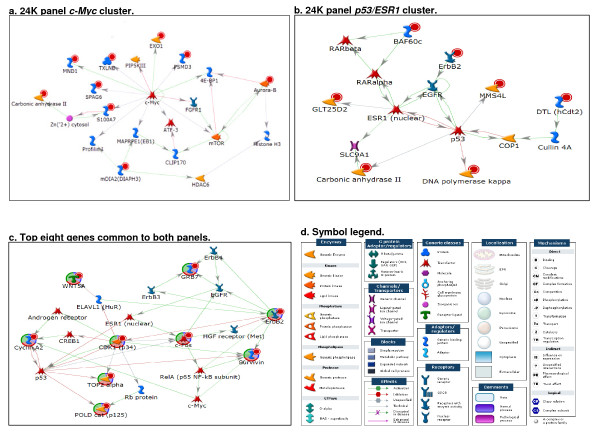
**MetaCore network analyses**. The top 47 genes that matched to the probes that were differentially expressed between HER2+ and HER2- samples (p-values < 0.01 and log2-fold change > |1.0|) in the 24K panel are enriched with genes (marked by red cycles) belonging to two distinct regulatory networks. **a**. The first network includes genes (marked by red cycles) functionally connected to *MYC *(*c-Myc*). **b**. The second network is enriched by genes (marked by red cycles) centered around *TP53 *(*p53*) and *ESR1*. **c**. The 8 genes differentially expressed in both the 24K and 1.5K panels (marked by red cycles) are all connected in a network that includes *TP53 (p53)*, *ESR1*, and *MYC *(*c-Myc*) **d**. Symbol legend. Abbreviations for gene names presented in Figure 5 are defined in Additional File 1, Tables S1-S3.

Table 3 lists the top 52 discriminating probes (representing 47 genes) from the 24K panel having p-values < 0.01 and log2-fold change > |1.0| between HER2+ and HER2- tumors. Network analysis of the 47 genes showed that several of these genes (indicated by red circles) are functionally linked to *v-myc avian myelocytomatosis viral oncogene homolog *(*MYC*), *tumor protein p53 *(*TP53*), and *estrogen receptor α *(*ESR1*) (Figure 5A-B). The top five discriminating genes from the 24K panel are *ERBB2*, *GRB7*, *per1-like domain-containing protein 1 *(*PERLD1*), *anti-silencing function 1, s. cerevisiae, homolog B *(*ASF1B*), and *chromosome 17 open reading frame 37 *(*C17ORF37*). Network analyses showed that the top eight discriminating genes common to both panels (indicated by red circles) are connected in a network built by the shortest path algorithm allowing two steps in the path (Figure 5C). The hubs of the networks include *MYC*, *TP53*, and *ESR1*.

**Table 3:** Probes from the 24K panel that had a log2-fold change > |1.0| and a t-test p-value < 0.01.

Symbol	p-value	log2-fold change
ERBB2*	0.0000001	1.458
ERBB2*	0.00004	2.017
*GRB7*	0.00008	1.851
*C17ORF37*	0.00009	1.131
*PERLD1*	0.00011	1.966
*GRB7*	0.00028	1.817
*ASF1B*	0.00041	1.306
*PAPSS2*	0.00057	1.257
*GRB7*	0.00091	2.119
*C13ORF3*	0.00095	1.073
*CDC6*	0.00099	1.756
*MND1*	0.00119	1.178
*C5ORF4*	0.00123	-1.203
*DUSP15*	0.00167	-1.456
*FAM54A*	0.00178	1.223
ERBB2*	0.00179	1.063
PSMD3*	0.00184	1.087
*KCND3*	0.00199	-1.359
*PLEKHB1*	0.00204	-1.133
*ITIH4*	0.00207	-1.062
SMARCD3 (BAF60C)*	0.00234	-1.122
*LOC441376*	0.00259	-1.966
*PLP1*	0.00297	-1.440
GLT25D2*	0.00301	-1.074
*MAPT*	0.00325	-1.352
CA2 (carbonic anhydrase II)*	0.00341	-1.446
*CCNE1*	0.00367	1.189
*VAX2*	0.00389	-1.229
TXLNB*	0.00395	-1.088
*NUSAP1*	0.00409	1.141
*OSTALPHA*	0.00420	2.421
*IGFN1*	0.00435	-1.251
*CEP55*	0.00448	1.211
DIAPH3*	0.00473	1.052
*CCDC48*	0.00521	-1.445
*CCDC153*	0.00551	-1.048
*PMP2*	0.00551	-1.185
*MGC20983*	0.00579	-1.247
AURKB*	0.00595	1.025
*FLJ25770*	0.00624	-1.259
EXO1*	0.00626	1.042
*HYDIN*	0.00643	-1.318
DTL*	0.00676	1.251
*ATP6V0A4*	0.00787	1.940
S100A7*	0.00787	3.327
*ARTN*	0.00824	-1.379
EME1 (MMS4L)*	0.00842	1.021
SPAG6*	0.00853	-2.443
POLQ (DNA polymerase kappa) *	0.00882	1.090
*ROBO2*	0.00941	-1.679
*PLP1*	0.00946	-1.190
*ZNF695*	0.01000	1.414

*Genes that cluster around *MYC*, *P53*, and *ESR1 *and indicated by red circles in Figure 5 a-b.

## Discussion

Gene expression profiling has created new possibilities for the molecular characterization of cancer. The resulting gene expression signatures have the potential to explain the genetic heterogeneity of breast cancer and allow treatment strategies to be planned in accordance with their probability of success in individual patients [2]. Molecular classification is changing the design of clinical trials. For example, the TAILORx http://www.cancer.gov/clinicaltrials/digestpage/TAILORx[28] and MINDACT http://www.eortc.be/services/unit/mindact/MINDACT_websiteii.asp[29] are two adjuvant breast cancer treatment trials in which patients are stratified according to select gene signatures present in their excised breast tumor. The molecular differences that underlie the phenotypes of breast cancer could reveal new therapeutic targets and influence clinical care [30].

To optimize the full capability of gene expression profiling using microarray-based assays, technologies are being optimized to reliably perform gene expression profiling on FFPE specimens, currently the most common type of clinical specimen available, particularly for phase III adjuvant treatment trials. FFPE is an extremely valuable resource of tissue for discovery and validation studies. While the combination of the Affymetrix GeneChip^® ^Human X3P Array (Santa Clara, Ca) and Arcturus Paradise™ system (Mountain View, CA) has been optimized for FFPE tissue, it has been the experience of other investigators [31] and ourselves (unpublished observations) that call rates are unacceptably low, typically less than 30% [31]. Whereas, we observed high call rates (percent of detectable genes at the p = 0.01 level), which are sample dependent, on average of > 87% and > 75% from the 1.5K and 24K panels, respectively (data not shown). Almac (Belfast, Ireland) has developed a promising technology that utilizes Affymetrix-based methodology and disease-specific arrays (DSAs) or transcriptome panels that have ~ 50,000 transcripts that can be utilized for FFPE tissue [32,33].

The present report describes gene expression analyses of FFPE using the DASL Assay from Illumina, designed specifically to profile degraded RNAs derived from FFPE tumor samples. The DASL Assay has a dynamic range of 2.5 to 3 logs and limit of detection of 1 × 10^4 ^molecules, parameters comparable to those determined using standard microarray molecular profiling [13]. Custom and commercially available gene panels have been successfully used on the DASL platform and resulting gene signatures have proven to have diagnostic value. A custom 512-gene panel was used to identify gene signatures that correlated with Gleason score and relapse of prostate cancer [34]. The 502 Cancer Panel^v1 ^and a 526 custom gene panel were used to identify gene expression patterns that were significantly associated with systemic progression after prostate specific antigen recurrence in men with prostate cancer [21]. The whole genome 24K gene panel for use with the DASL platform recently became commercially available [16].

Our objective was to compare the performance of the 1.5K panel to the more recent 24K panel using the DASL platform to determine whether genes behave similarly between gene panels with different densities. The high correlations (0.815-0.997) observed between technical and extract replicates for both gene panels demonstrate that the reproducibility of results from both the 1.5K and 24K gene panels was excellent. The 24K panel revealed less variation between both technical and extract replicates compared to the 1.5K panel. Although it may be expected that the variability of hybridization signal intensities would be less for the 1.5K panel due to the higher probe density per gene for the 1.5K compared to the 24K panel, the 24K panel has a more stringent array hybridization condition compared to the 1.5K panel (i.e., the length of the probes is 50 nucleotides for the 24K BeadArray compared to ~22 nucleotides for the 1.5K panel). In addition, most of the genes on the 1.5K array are cancer-related and thus, in our study were expressed at higher levels compared to the genes on the 24K array. Furthermore, the intensity for the 1.5K array is the sum of a dual color assay (cy3+cy5 channels), whereas the 24K assay is a single-color assay (cy3), the hybridization conditions and washes are different, and the readouts are different (Universal Array Matrix versus whole genome BeadChip) and therefore, the scan settings are different. Lastly, it should be recognized that these technologies measure relative expression within the context of each platform.

As only 17 probes are identical of the 498 common genes, the two platforms have mostly non-overlapping nucleotide sequences for the same transcript target. The targeted regions in the 24K assay were designed to correspond to the largely 3' biased 50 nucleotide probe sequence content of the HumanRef-8 v3 BeadChip [16] and the targeted regions of the 1.5K assay were not restricted to the 3' end of transcripts [13]. Specific probe information can be found online at http://www.switchtoi.com/annotationfiles.ilmn[35]. For genes with poor fold-change correlations, it is also conceivable that the probes may be identifying splice variants of the same gene, and thereby targeting different mRNA isoforms due to variations in probe position on the panels.

At the gene level, we observed larger median correlations between the 1.5K and 24K panels for genes that were represented by more probes. In addition, within-platform data for the 1.5K assay, the expression profiles generated with three probes/transcript correlated well (R^2^~0.99) with those profiles generated with four or more (up to ten) probes/transcript [13].

The inter-panel agreement was good for probes with sequences that matched across the 1.5K and 24K panels; correlations ranged from 0.652 to 0.899. However, the agreement for probes that had different sequences that mapped to the same gene had fair correlation across the two panels; correlations ranged from 0.485 to 0.573. This is not unexpected as the expression level appears to be a function of the probe sequence location within the gene such that different probe sequences may correspond to different cDNA synthesis efficiencies and different oligo hybridization efficiencies [23]. This was particularly evident for the *ERBB2 *gene expression obtained from the 24 K panel (Figure 3). It has been suggested that the differences in expression values between the two panels could result from non-specific hybridization in the 1.5K array (since increase in stringency in the hybridization affects the intensity of expression values) or from the increased complexity of the labeling step in the 24 K array that may lead to "less" labeling and reduced hybridization. However, hybridization conditions for both platforms have been optimized for the different length of probe (~22 vs. 50 nucleotides) minimizing non-specific/cross-hybridization. Also, the short address codes for the 1.5K array were carefully selected to have a similar overall length, GC-content, and melting temperature (T_m_), whereas for the 24K array the targeted regions were somewhat restrained having been pre-determined by the 50 nucleotide probe sequences on the whole-genome gene expression BeadChip (HumanRef-8 v3). Despite the differences in absolute intensity, the relative differences between the HER2+ and HER2- groups is conserved across both platforms and all six probes.

It is also important to note that because of differences between the two platforms [e.g., non-overlapping nucleotide sequences for the same transcript targets as well as different hybridization conditions for the 1.5K and 24K assays (as described above)], direct comparisons of the raw intensities will yield seemingly poor cross-platform correlations. However, fold-change correlations of the gene intensities between the two platforms provide a common metric for comparisons.

Both panels detected significant differential *ERBB2 *gene expression between HER2+ and HER2- breast tumors, and the HER2 gene was the most differentially expressed gene for both panels. These results indicate that both panels correctly classified the HER2 status of the tumors when comparing gene expression to protein expression determined by IHC (gold standard) and when considering IHC score of 0-1+ as HER2- and IHC scores of 2-3+ as HER2+. The two tumors that had an IHC score of 2+ as defined by the 2007 ASCO/CAP guidelines [19] were initially considered 3+ when using the FDA-approved guidelines [20]. In addition, there were eight concordant genes across the panels that had a log2-fold change > |0.5| and p-value < 0.05 to differentiate between HER2+ and HER2- tumors. Two of these 8 genes, *ERBB2 *and *GRB7*, are in the 10-gene *HER2 *cluster observed by Perou and Sorlie [4,5]. We selected tumors to closely match on hormone receptor (majority are positive) and nodal status (all node positive) to maximize the difference in gene signatures largely resulting from the HER2 phenotype. We also wanted to minimize the molecular heterogeneity that can be found in HER2+ tumors, influenced by the hormone receptor status and basal-type signatures [36,37]. Several well-known gene signatures identifying the same population of patients have very few genes in common, a feature of complex gene-expression data that contain large numbers of highly correlated variables (i.e., gene-expression measurements) [30]. Several different combinations of the correlated variables can be selected to build similarly accurate prediction models. Thus, different differential gene lists from various platforms can be considered comparable when they reveal similar biological functions [38].

As the main purpose of gene expression studies using microarrays is to reveal the underlying biological differences between groups, functional networks were generated using MetaCore. We observed that the top 52 discriminating probes from the 24K panels are enriched with genes functionally linked to *MYC *and *TP53/ESR1 *networks. Nine of the 10 genes in the *HER2 *gene cluster from the Perou/Sorlie dataset [4,5] form a regulatory network also centered around *TP53 *and *ESR1*. In addition, four (*ERBB2*, *GRB7*, *PERLD1*, and *C17ORF37*) of the top five HER2 discriminating genes from the 24K panel are genes commonly amplified in the *HER2 *amplicon (17q12-q21) and were overexpressed in HER2+ tumors. Their gene expressions were also highly correlated (r^2 ^= 0.806-0.912, p < 0.005). Lastly, network analyses showed that the top eight discriminating genes common to both panels are connected by the shortest path network analysis with a two-step extension. Interconnecting genes include *c-Myc (MYC)*, *TP53*, and *ESR1*.

Thus, it appears that genes in the *MYC*, *TP53*, and *ESR1 *regulatory networks are important in differentiating between HER2-positive and -negative tumors. HER2 expression has been shown to be influenced by the presence of ESR1 [36,37,39-42]. Although we selected tumors positive for the estrogen receptor protein (ER+) by immunohistochemistry, 11 of 13 HER2 0-2+ tumors had high *ESR1 *expression (≥ 12), whereas only two of the seven HER2 3+ tumors had high *ESR1 *expression (Fisher's Exact p = 0.022). In addition, significant correlations between *ESR1 *gene expression and ER protein expression levels were observed for the 1.5K (r^2 ^= 0.71; p = 0.002) and 24K (r^2 ^= 0.65; p = 0.006) panels (Additional File 1, Figure S4). Overall, the network analysis demonstrated biological consistency between the gene panels. Our data are consistent with recent findings that demonstrated that highly consistent biological information can be generated from different microarray platforms [38]. As this study was designed primarily to evaluate and compare the technical performances of the two platforms with pre-defined tumor selection (e.g., all ER+ and node-positive tumors), conclusions regarding clinically relevant information of HER2+/HER2- biology need to be further validated.

## Conclusions

Our results indicate that the relative gene expression intensities are highly correlated and biological consistency is observed between two different density gene panels when analyzed using the DASL technology. These findings suggest that the 1.5K and 24K panels are both adequate platforms for gene expression profiling of FFPE tumors. The 24K panel is ideally suited for whole genome screening/discovery studies, whereas, the 1.5K panel is suitable for cancer-focused studies. Screening of the 24K panel is also an appropriate approach to identify smaller, promising gene signatures, which when validated, can be utilized in clinical testing.

Our results and previous findings, taken together, demonstrate that the DASL assay provides a reliable approach to gene expression profiling in FFPE tumors. Several reports have already shown that gene signatures arising from the DASL assay have prognostic potential. A promising direction of research is to examine the hypothesis that different markers and biologic pathways may be involved in determining prognosis, response, and resistance to therapy in different molecular subgroups of breast cancers [30]. As ever-larger clinical data sets become available for gene-expression analysis, the DASL assay using FFPE tissue will help develop predictors of molecular class-specific prognosis and treatment response. This will allow for detailed investigations of gene pathways and interactions indicated by the resultant gene signatures that are truly predictive of clinical endpoints to better understand the biology underlying the disease [43-46]. Lastly, the combination of multiple forms of molecular data (protein- and gene-based) and clinical and demographic factors has the potential to identify unique characteristics of the individual and lead to more effective customized health care strategies [47,48].

## Competing interests

Jian-Bing Fan, Craig April, and Eliza Wickham-Garcia are employees and/or stockholders of Illumina Inc, where the DASL technology was developed. No other authors have any competing interests.

## Authors' contributions

MMR Identified the cases from Mayo Clinic Tissue Registry, developed the IRB protocol, and had overall responsibility for the project. MMR made substantial contributions to the conception and design of the study and to the interpretation of the data. MMR also drafted the manuscript and has given final approval of the manuscript. MMR received NIH funding to support this study. JEP made substantial contributions to the conception and design of the study and to the analysis and interpretation of the data. JEP also critically revised the manuscript and has given final approval of the manuscript. SKA made substantial contributions to the conception and design of the study and to the analysis and interpretation of data. SKA also created the figures and has given final approval of the manuscript. YAW Performed the network analyses and included these in the manuscript and has given final approval of the manuscript. MAZ Performed the RNA isolations and checked quality of the RNA. MAZ made contributions to the interpretation of data and gave final approval of the manuscript. ALO made substantial contributions to the conception and design of the study and to the analysis and interpretation of the data. ALO also critically revised the manuscript and has given final approval of the manuscript. AEM performed central scoring of HER2 immunohistochemistry on TMA sections. AEM made contributions to the interpretation of data and gave final approval of the manuscript. ACD Made substantial contributions to the conception and design of the study and to the analysis and interpretation of the data. ACD also critically revised the manuscript and has given final approval of the manuscript. BC performed central scoring of HER2 immunohistochemistry on whole sections. BC made contributions to the interpretation of data and gave final approval of the manuscript. CSA made substantial contributions to the conception and design of the study and to the analysis and interpretation of the data. CSA carried out the whole genome DASL analysis, critically reviewed the manuscript, and has given final approval of the manuscript. EW-G made substantial contributions to the analysis and interpretation of the data. EW-G assisted in the whole genome DASL analysis, critically reviewed the manuscript, and has given final approval of the manuscript. RBJ made substantial contributions to the conception and design of the study and to the analysis and interpretation of the data. RBJ also critically reviewed the manuscript and has given final approval of the manuscript. JMC made substantial contributions to the conception and design of the study and to the analysis and interpretation of the data. The 1.5K DASL analysis was carried out under the direction of JMC. JMC also critically revised the manuscript and has given final approval of the manuscript. JJ made substantial contributions to the conception and design of the study and to the analysis and interpretation of the data. JJ also critically revised the manuscript and has given final approval of the manuscript. EAP made substantial contributions to the conception and design of the study and to the analysis and interpretation of the data. EAP also critically reviewed the manuscript and has given final approval of the manuscript. J-BF made substantial contributions to the conception and design of the study and to the analysis and interpretation of the data. The whole genome DASL analysis was carried out under the direction of J-BF. J-BF also critically revised the manuscript and has given final approval of the manuscript. WLL Made substantial contributions to the conception and design of the study and to the analysis and interpretation of the data. WLL is the director of the laboratories where the tissue sectioning and RNA extractions occurred. WLL also critically reviewed the manuscript and has given final approval of the manuscript.

## Authors Information

M.M.R. (translational science researcher and PI of study)

J.E.P. (statistician)

S.K.A. (statistician)

Y.A.W. (bioinformatician)

M.A.Z. (technician)

A.L.O. (statistician)

A.E.M. (pathologist)

A.C.D. (statistician)

B.C. (pathologist)

C.S.A. (researcher at Illumina)

C.S.A. (researcher at Illumina)

E.W-G. (researcher at Illumina)

R.B.J. (Director of the Mayo Clinic Clinical Cytogenetics Laboratory)

J.M.C. (Director of the Mayo Clinic Cancer Center Genotyping Shared Resource)

J.J. (Director of the Mayo Clinic Cancer Center Microarray Shared Resource)

E.A.P. (co-PI of NIH funding)

J-B.F. (researcher and study lead at Illumina)

W.L.L. (Director of the Mayo Clinic Cancer Center TACMA Shared Resource)

## Pre-publication history

The pre-publication history for this paper can be accessed here:

http://www.biomedcentral.com/1755-8794/3/60/prepub

## Supplementary Material

Additional file 1 **Figures S1-S4 and Tables T1-T3**. Additional Figures S1-S3 are additional figures (S1-S2) that explain the associations between 1) block procurement year and RP13a qPCR Cq, 2) block procurement year and scanner p95 readings, and 3) RP13a and qPCR Cq and scanner p95 readings. An additional figure (S3) explains the correlation between gene panels according to number of probes per 24K gene symbol. Additional Figure S4 explains the relationship between ER immunohistochemical staining and *ESR1 *gene expression intensity. Additional Tables T1-T3 define the gene symbols presented in Figure 5. Click here for file
