# Supplementary material for: Expression profiling of formalin-fixed paraffin-embedded primary breast tumors using cancer-specific and whole genome gene panels on the DASL® platform
Source: BMC Med Genomics. 2010 Dec 20;3:60. doi: 10.1186/1755-8794-3-60 (PMC3022545; doi:10.1186/1755-8794-3-60)
Supplement: Additional file 1 — Figures S1-S4 and Tables T1-T3. Additional Figures S1-S3 are additional figures (S1-S2) that explain the associations between 1) block procurement year and RP13a qPCR Cq, 2) block procurement year and scanner p95 readings, and 3) RP13a and qPCR Cq and scanner p95 readings. An additional figure (S3) explains the correlation between gene panels according to number of probes per 24K gene symbol. Additional Figure S4 explains the relationship between ER immunohistochemical staining and ESR1 gene expression intensity. Additional Tables T1-T3 define the gene symbols presented in Figure 5. [file 1755-8794-3-60-S1.DOC]

**Additional Files**

**Reinholz et al., BMC Medical Genomics 1613985825360642**

**Expression profiling of formalin-fixed paraffin-embedded primary breast tumors using cancer-specific and whole genome gene panels on the DASL® platform**

Monica M. Reinholz^1*^, Jeanette E. Eckel-Passow^2^, S. Keith Anderson^2^, Yan W. Asmann^2^, Michael A. Zschunke^1^, Ann L. Oberg^2^, Ann E. McCullough^3^, Amylou C. Dueck^4^, Beiyun Chen^1^, Craig S. April^5^, Eliza Wickham-Garcia^5^, Robert B. Jenkins^1^, Julie M. Cunningham^1^, Jin Jen^6^, Edith A. Perez^7^, Jian-Bing Fan^5^, and Wilma L. Lingle^1^

^1^Department of Laboratory Medicine and Pathology, Mayo Clinic, 200 First St SW, Rochester, Minnesota, 55905, USA; ^2^Division of Biomedical Statistics and Informatics Mayo Clinic, 200 First St SW, Rochester, Minnesota, 55905, USA; ^3^Department of Pathology, Mayo Clinic, 13400 E. Shea Blvd, Scottsdale, Arizona, 85259, USA; ^4^Section of Biostatistics, 13400 E. Shea Blvd, Scottsdale, Arizona, 85259, USA; ^5^Department of Scientific Research, Illumina Inc., 9885 Towne Centre Drive, San Diego, California, 92121, USA; ^6^Division of Pulmonary and Critical Care Medicine, Mayo Clinic, 200 First St SW, Rochester, Minnesota, 55905, USA; ^7^Division of Hematology and Oncology, Mayo Clinic, 4500 San Pablo Road, Jacksonville, Florida, 32224, USA

**Additional File 1, Figure S1. Associations between block procurement year and RP13a qPCR Cq and scanner P95 readings for the 1.5K DASL panel.**

No significant associations were observed between the year the block was procured and the RP13a qPCR Cq values (slope: -0.34; p=0.0537) **(Additional Figure S1A)** or with the scanner P95 readings (slope: 0.008; p=0.516) **(Additional Figure S1B)**.

**S1A.**

**S1B.**

**Additional File 1, Figure S2. Association between RP13a qPCR Cq and scanner P95 readings for the 1.5K DASL panel.**

No significant association was observed between RP13a qPCR Cq values and the scanner P95 readings [slope: 0.00018 (p=0.482].

**Additional File 1, Figure S3. Correlation between gene panels according to number of probes per 24K gene symbol.**

**Additional Figure S3** shows that at the gene level, larger correlations between the 1.5K and 24K panels were observed for genes that were represented by more probes. For the 498 genes in common across the two platforms, the 1.5K panel had 3 probes representing each gene. However, the 24K panel had 333 genes that were represented by a single probe, 86 that were represented by two probes, 64 that were represented by three probes, and 15 that were represented by four-six probes. The average expression was calculated for each platform for all 498 gene symbols in common. The median Pearson correlation of expression levels across the gene panels were 0.36 (1^st^/3^rd^ quartiles: 0.1843/0.5990), 0.46 (1^st^/3^rd^ quartiles: 0.2404/0.6517) , 0.49 (1^st^/3^rd^ quartiles: 0.2323/0.6893), and 0.53 (1^st^/3^rd^ quartiles: 0.3915/0.6102) for genes having one, two, three, and four to six probes per gene on the 24K panel, respectively.

**Additional File 1, Figure S4. Relationship between ER immunohistochemical staining and *ESR1* gene expression intensity.** Protein levels (percent of cells staining positive for ER by immunohistochemistry) correlated well with mRNA levels as measured by DASL. **Additional Figures S4A and S4B** show the relationship of the ER-staining to the *ESR1* expression values for each of the two platforms. For the 16 samples that had ER staining data available, within the 1.5K and 24K platforms the Pearson correlation values were 0.71 (p=0.002) and 0.65 (p=0.006), respectively, comparing the ER staining to the *ESR1* expression levels. The circles are color coded red, blue, green, and black based on ER staining of 100%, 99-90%, 89-60%, and less than 60%, respectively. Specifically, **Additional Figure S4A** shows the 1.5K platform *ESR1* expression versus ER staining (slope: 0.035, p=0.002) and **Additional Figure S4B** shows the 24K platform *ESR1* expression versus ER staining (slope: 0.021, p=0.007). **Additional Figure S4C** shows the correlation of *ESR1* expression levels between both platforms for the samples that have ER staining values (Pearson correlation= 0.96; p<0.0001, note: the identity line is shown for reference only).

**S4A. *ESR1* 1.5K vs. ER Staining**

**S4B. *ESR1* 24K vs. ER Staining**

***ESR1* 1.5K**

***ESR1* 24K**

**ER Staining**

**(% cells staining positive for ER by IHC)**

**ER Staining**

**(% cells staining positive for ER by IHC)**

**S4C. *ESR1* 24K vs. *ESR1* 1.5K**

***ESR1* 24K**

***ESR1* 1.5K**

**Additional File 1, Table S1. Names for gene abbreviations presented in Figure 5a of main manuscript**.

| **Gene Abbreviation** | **Gene Name** |
| --- | --- |
| *EXO1* | *exonuclease 1* |
| *PSMD3* | *proteasome (prosome, macropain) 26S subunit, non-ATPase, 3*; |
| *4E-BP1* | *eukaryotic translation initiation factor 4E binding protein* 1 |
| *aurora-B* | *aurora kinase B* |
| *histone h3* | *H3 histone, family 3A* |
| *HDAC*: | *histone deacetylase 6* |
| *mDIA2(DIAPH3)* | *diaphanous homolog 3* (Drosophila) |
| *Profilin I* | *profilin I* |
| *Carbonic anhydrase II* | *Carbonic anhydrase II* |
| *MND1* | *meiotic nuclear divisions 1 homolog* (S. cerevisiae) |
| *TXLNB* | *taxilin beta* |
| *PIP5KIII* | *phosphoinositide kinase FYVE finger containing* |
| *c-Myc* | *v-myc myelocytomatosis viral oncogene homolog* (avian) |
| *FGFR1* | *fibroblast growth factor receptor 1* |
| *mTOR* | *mechanistic target of rapamycin* (serine/threonine kinase) |
| *CLIP170* | *CAP-GLY domain containing linker protein 1* |
| *ATF-3* | *activating transcription factor 3* |
| *MAP* | *microtubule-associated protein* |
| *RPE1(EB1)* | *RP/EB family, member 1* |
| *S100A7* | *S100 calcium binding protein A7* |
| *SPAG6* | *sperm associated antigen 6* |

**Additional File 1, Table S2. Names for gene abbreviations presented in Figure 5b of main manuscript.**

| **Gene Abbreviation** | **Gene Name** |
| --- | --- |
| *RARbeta* | *retinoic acid receptor, beta* |
| *RARalpha* | *retinoic acid receptor alpha* |
| *BAF60c* | *SWI/SNF related, matrix associated, actin dependent regulator of chromatin, subfamily d, member 3* |
| *ErbB2 v-erb-b2* | *erythroblastic leukemia viral oncogene homolog 2, neuro/glioblastoma derived oncogene homolog* (avian) |
| *EGFR* | *epidermal growth factor receptor (erythroblastic leukemia viral (v-erb-b) oncogene homolog* (avian) |
| *ESR (nuclear)* | *estrogen receptor 1* |
| *GLT25D2* | *glycosyltransferase 25 domain containing 2* |
| *MMS4L* | *essential meiotic endonuclease 1 homolog 1* (S. pombe) |
| *p53* | *tumor protein p53* |
| *COP1* | *ring finger and WD repeat domain 2* |
| *Cullin 4A* | *Cullin 4A* |
| *DTL (hCdt2)* | *denticleless homolog* (Drosophila) |
| *SLC9A1* | *solute carrier family 9 (sodium/hydrogen exchanger), member 1* |
| *Carbonic anhydrase II* | *Carbonic anhydrase II* |
| *DNA polymerase kappa* | *polymerase (DNA directed) kappa* |

**Additional File 1, Table S3.** **Names** **for gene abbreviations presented in Figure 5c of main manuscript.**

| **Gene Abbreviation** | **Gene Name** |
| --- | --- |
| *ErbB4* | *v-erb-a erythroblastic leukemia viral oncogene homolog 4* (avian) |
| *GRB7* | *growth factor receptor-bound protein 7* |
| *ErbB2* | *v-erb-b2 erythroblastic leukemia viral oncogene homolog 2, neuro/glioblastoma derived oncogene homolog* (avian) |
| *EGFR* | *epidermal growth factor receptor* |
| *ErbB3* | *v-erb-b2 erythroblastic leukemia viral oncogene homolog 3* (avian) |
| *c-Fos* | *FBJ murine osteosarcoma viral oncogene homolog* |
| *HGF receptor* (*Met*) | *met proto-oncogene* (*hepatocyte growth factor receptor*) |
| *Survivin* | *baculoviral IAP repeat-containing 5* |
| *RelA* (*p65 NF-kB subunit*) | *v-rel reticuloendotheliosis viral oncogene homolog A* (avian) |
| *c-Myc* | *v-myc myelocytomatosis viral oncogene homolog* (avian) |
| *ESR1* (nuclear) | *estrogen receptor 1* |
| *WNT5A* | *wingless-type MMTV integration site family, member 5A* |
| *ELAVL1* (HuR) | *ELAV* (*embryonic lethal, abnormal vision, Drosophila*)*-like 1* (Hu antigen R) |
| *CREB1* | *cAMP responsive element binding protein 1* |
| *CDK1* (*p34)* | *cyclin-dependent kinase 1* |
| *TOP2 alpha* | *topoisomerase* (*DNA*) *II alpha 170kDa* |
| *Rb Protein* | *retinoblastoma 1* |
| *POLD cat* (*p125*) | *polymerase* (*DNA directed*) *delta 1 catalytic subunit 125kDa* |
| *p53*: | *tumor protein p53* |
| *Cyclin A2* | *cyclin A2* |
| *Androgen receptor* | *androgen receptor* |
